# Phylogenetic Analysis of the Membrane Attack Complex/Perforin Domain-Containing Proteins in *Gossypium* and the Role of *GhMACPF26* in Cotton Under Cold Stress

**DOI:** 10.3389/fpls.2021.684227

**Published:** 2021-11-19

**Authors:** Pengyun Chen, Hongliang Jian, Fei Wei, Lijiao Gu, Tingli Hu, Xiaoyan Lv, Xiaohao Guo, Jianhua Lu, Liang Ma, Hantao Wang, Aimin Wu, Guangzhi Mao, Shuxun Yu, Hengling Wei

**Affiliations:** ^1^State Key Laboratory of Cotton Biology, Institute of Cotton Research of Chinese Academy of Agricultural Sciences, Anyang, China; ^2^Forest Department, Forestry College, Hebei Agricultural University, Baoding, China; ^3^College of Life Sciences, Xinyang Normal University, Xinyang, China

**Keywords:** *Gossypium*, membrane attack complex/perforin (MACPF), cold stress, VIGS, WGCNA

## Abstract

The membrane attack complex/perforin (MACPF) domain-containing proteins are involved in the various developmental processes and in responding to diverse abiotic stress. The function and regulatory network of the *MACPF* genes are rarely reported in *Gossypium* spp. We study the detailed identification and partial functional verification of the members of the MACPF family. Totally, 100 putative MACPF proteins containing complete MACPF domain were identified from the four cotton species. They were classified into three phylogenetic groups and underwent multifold pressure indicating that selection produced new functional differentiation. Cotton *MACPF* gene family members expanded mainly through the whole-genome duplication (WGD)/segmental followed by the dispersed. Expression and *cis-*acting elements analysis revealed that MACPFs play a role in resistance to abiotic stresses, and some selected *GhMACPFs* were able to respond to the PEG and cold stresses. Co-expression analysis showed that *GhMACPFs* might interact with valine-glutamine (VQ), WRKY, and Apetala 2 (AP2)/ethylene responsive factor (ERF) domain-containing genes under cold stress. In addition, silencing endogenous *GhMACPF26* in cotton by the virus-induced gene silencing (VIGS) method indicated that *GhMACPF26* negatively regulates cold tolerance. Our data provided a comprehensive phylogenetic evolutionary view of *Gossypium* MACPFs. The MACPFs may work together with multiple transcriptional factors and play roles in acclimation to abiotic stress, especially cold stress in cotton.

## Introduction

The membrane attack complex/perforin (MACPF) proteins are pore-forming proteins across the cellular membrane in plants and other organisms. They have a signature motif, Y/S-G-G/S-H-X7-G-G, and play important roles in mammalian immunity and bacterial pathogenesis (Hadders et al., 2007; Wade and Tweten, 2015; Johnson et al., 2017; Moreno-Hagelsieb et al., 2017; Ni and Gilbert, 2017; Yu et al., 2020). In *Arabidopsis*, the necrotic spotted lesion 1 (NSL1) protein, containing a putative MACPF domain, was involved in the negative regulation of programmed cell death (PCD) and defense response (Noutoshi et al., 2006). Moreover, loss function of the MACPF domain could trigger cell death through activating salicylic acid (SA) signaling in *Arabidopsis* (Morita-Yamamuro et al., 2005; Fukunaga et al., 2017).

Environmental stress is a major challenge in agricultural production, affecting plant photosynthesis efficiency, cell membrane homeostasis, and plant growth and development (Hasegawa et al., 2000; Odukoya et al., 2019). Plants have developed sophisticated physiological and biochemical adaptation that enables them to survive through the regulation of multiple hormones including salicylic acid (SA), ethylene, and abscisic acid (ABA) (Zhu, 2002; Vlot et al., 2009; Hasanuzzaman et al., 2017; Emamverdian et al., 2020). SA exerts key effects in disease resistance by modulating host cell death and defense gene expression (Vlot et al., 2009). An SA-responsive protein is known as constitutively activated cell death 1 (CAD1) has been found to have a conservative MACPF domain. The *CAD 1* mutant altered endophytic phyllosphere microbiota and displays leaf tissue damage in *Arabidopsis*. CAD1 is important for plant defense and likely negatively regulates plant immunity (Morita-Yamamuro et al., 2005; Tsutsui et al., 2006; Chen T. et al., 2020).

In recent years, different transcriptomic data showed that the *MACPF* genes were involved in growth, development, and response to abiotic stresses in sorghum, rice, and maize (Yu et al., 2020). However, the evolution and function of the *MACPF* genes in *Gossypium* spp. were still unknown. The underlying interaction among the *MACPFs* and other genes was also unclear. The weighted gene co-expression network analysis (WGCNA) was a useful method to identify the potential network through the transcriptomic analysis, which had been used to study development and abiotic stress in many core traits (Chen X. et al., 2020; Cheng et al., 2021).

As a pioneer crop in the barren land, cotton has a natural stress resistance through the complex genome structure (Li et al., 2015). Understanding the potential function of the MACPFs in cotton will be helpful to the cotton breeding and functional analysis. In this study, we performed a genome-wide identification of the MACPF family members in tetraploid species *Gossypium hirsutum* (AD1) and *Gossypium barbadense* (AD2) and their ancestor diploid *Gossypium arboreum* (A2) and *Gossypium raimondii* (D5) (Paterson et al., 2012; Du et al., 2018; Hu et al., 2019). The evolutionary relationships of the MACPFs were investigated and the putative interactions of the *MACPFs* with other genes were analyzed by the WGCNA. In total, 38 *GhMACPFs*, 33 *GbMACPFs*, 14 *GaMACPFs*, and 15 *GrMACPFs* were identified from the four *Gossypium* spp. genomes. Phylogenetic, conserved structural motif, the whole-genome duplication (WGD), non-synonymous substitution (Ka), and synonymous substitution (Ks) showed that the *MACPFs* were subjected to functional gene selection. Co-expression indicated that *GhMACPFs* played an important role in responding to abiotic stress. and Apetala 2 (AP2)/ethylene responsive factor (ERF), valine-glutamine (VQ), and WRKY transcription factors (TFs) were the co-expression genes of *GhMACPFs* in the regulation of cold stress response. In addition, silencing of *GhMACPF26* in cotton enhanced the tolerance to cold stress. This study will lay a solid foundation for further exploration of the functions of *GhMACPFs* in cotton.

## Materials and Methods

### Identification and Molecular Characterization of the MACPF Domain Family in Multiple Plants

A total of 15 genome databases, which contained Arabidopsis thaliana (L.) Heynh., Oryza sativa Linn., Solanum lycopersicum Linn., Vitis vinifera Linn., Ananas comosus (L.) Merr., Amborella trichopoda Baill., Carica papaya Linn., Theobroma cacao Linn., G. hirsutum, G. barbadense, Gossypium arboreum, G. raimondii, Brassica napus Linn., Brassica oleracea Linnaeus, and Brassica rapa Linn., were obtained from the CottonGen^1^ (Yu et al., 2014), the Joint Genome Institute (JGI) database^2^ (Goodstein et al., 2012), and the National Center for Biotechnology Information (NCBI) database^3^ (Sayers et al., 2010), respectively. Four AtMACPF genes, including AtMACPF01 (AT1G14780), AtMACPF02 (AT1G28380), AtMACPF03 (AT1G29690), and AtMACPF04 (AT4G24290), were used as query sequences to blast the selected proteins database via the protein-protein BLAST (BLASTP) method (E-value = 1 × 10^–3^). The hidden Markov model (HMM) profile of the MACPF domain (Pfam01823) was downloaded from the Pfam web^4^ (El-Gebali et al., 2019) and the Hmmsearch (version 3.2.1) software (Eddy, 2011) was used to scan the 14 genome proteins. In addition, the web of the SMART database^5^ (Ivica et al., 2009) and the NCBI conserved domain database^6^ (Marchlerbauer et al., 2015) were used to confirm the conservative MACPF domain. Finally, we identified the molecular characterization through the Softberry web^7^ and the ExPASy website^8^.

### Phylogenetic and Collinearity Analysis of the *MACPF* Genes

The Multiple Alignment using Fast Fourier Transform (MAFFT) (version 7.4.0.7) (Katoh and Standley, 2014), the Gblocks (version 0.91), and the PhyML^9^ (Guindon et al., 2005) were used to align 184 MACPF domain-containing proteins, analyze the conserved site sequences, and construct the maximum likelihood (ML) tree, respectively. R/ggtree (Yu et al., 2016) was applied to color the final tree file.

The MCScanX software (Wang et al., 2012) was used to analyze gene collinearity with default parameter among in *Gossypium* spp., which contained *G. hirsutum* and *G. barbadense*; *G*. *arboreum*, *G*. *raimondii*, and *G. hirsutum*; and *G*. *arboreum*, *G*. *raimondii*, and *G. barbadense*. The duplication event was implemented through the commands of the MCScanX/duplicate_gene_classifier code. To determine selection pressure, the BLASTP (Mark et al., 2008), the ParaAT (Zhang et al., 2012), and the KaKs_Calculator (version 2.0) (Wang et al., 2010) were used to calculate the Ka and Ks values.

### Gene Location, *Cis* Element, and Conserved Motif Analysis

The MACPF domain-containing gene locations were obtained from the general feature format (GTF), which contained gene id, source, feature, start, end, and score via the python script. The intron/exon structure of the *MACPF* genes was extracted from the GTF. The conserved motifs in the MACPF proteins were obtained by submitting the protein sequences to the MEME web^10^ (Bailey et al., 2009) with the default of 30 amino acids in length. Gene structure view of the TBtools software (Chen C. et al., 2020) was used to integrate the motif and gene structure. Finally, the *cis-*elements of the promoter (2 kb) in the *Gossypium MACPF* genes were analyzed through the PlantCARE web^11^ (Lescot et al., 2002).

### Expression Profiles and Construction of Co-expression Network

The transcripts of the *MACPF* genes in different tissues and multiabiotic stresses were collected from previous research (PRJNA490626) (Hu et al., 2019). Transcriptomic analysis was performed according to the previously described protocols (Chen P. et al., 2020). Differentially expressed genes (DEGs) were analyzed by using the R/edgeR package (Robinson et al., 2010). The R/WGCNA package (Langfelder and Horvath, 2008) was used to identify the co-expression module and construct the co-expression networks. In addition, *hub* genes were identified via the cytoHubba/Cytoscape software (Shannon et al., 2003; Chin et al., 2014) and the final network was showed by the Cytoscape.

### Plant Cultivation, RNA Isolation, and Quantitative Real-Time-PCR Analysis

The Texas Marker-1 (TM-1) cotton seeds were provided by the Institute of Cotton Research of the Chinese Academy of Agricultural Sciences and planted in artificial growth conditions under a photoperiod of 16 h light/8 h darkness at 28/22°C. In order to verify the results of the transcriptome, the cotton plants grown at the three-leaf stage were subjected to stress treatment. For the drought treatment, the seedlings of TM-1 were irrigated with 400 mM polyethylene glycol 600 (PEG 600) for 0, 1, 6, 12, and 24 h. For the cold stress, the seedlings were irrigated with continuous cold stress (4°C) in a plant incubator/illumination incubator for 0, 2, 4, 6, 8, 12, and 24 h. Leaf samples were collected from five uniform plants, then quickly frozen in liquid nitrogen and stored at −80°C. All the experiments were repeated three times.

Total RNA was isolated by using the RNAprep Pure Plant Kit (Polysaccharides and Polyphenolics-rich, DP441) (TIANGEN, Beijing, China). The Mir-X^TM^ miRNA First-Strand Synthesis Kit (Takara Biotechnology Corporation Ltd., Dalian, China), the SYBR Green PCR Supermix Kit (Bio-Rad Laboratories), and the 7500 Real-Time PCR System (Applied Biosystems) were used to synthesize complementary DNA (cDNA) and analyze gene expression levels, respectively. *GhUBQ7* (NCBI accession: DQ116441) (Tu et al., 2007) was used as an endogenous control. All the primers designed by Primer 6 were listed in Supplementary Table 1.

### Virus-Induced Gene Silencing and Cold Stress

To silence *GhMACPF26* in cotton, a 300-bp fragment of the gene was PCR amplified and cloned into a pTRV2 vector; the primers used for implication were listed in Supplementary Table 1. A specific fragment of *GhMACPF26* was amplified from the upland cotton accession of the TM-1 cDNA library. The constructs were transformed into *Agrobacterium tumefaciens* strain LBA4404. The virus-induced gene silencing (VIGS) method and the growth condition of the TM-1 cotton seedlings were described previously by Gu et al. (2018). The VIGS and control plants were treated at 4°C for 24 h and the leaves were sampled for biochemical experimental analysis. Malondialdehyde (MDA) was measured as the manual of the MDA assay kit [thiobarbituric acid (TBA) method]. Each experiment was performed in triplicate.

## Results

### Membrane Attack Complex/Perforin Domain Proteins in the Allotetraploid and Diploid Cotton

**FIGURE 1:**
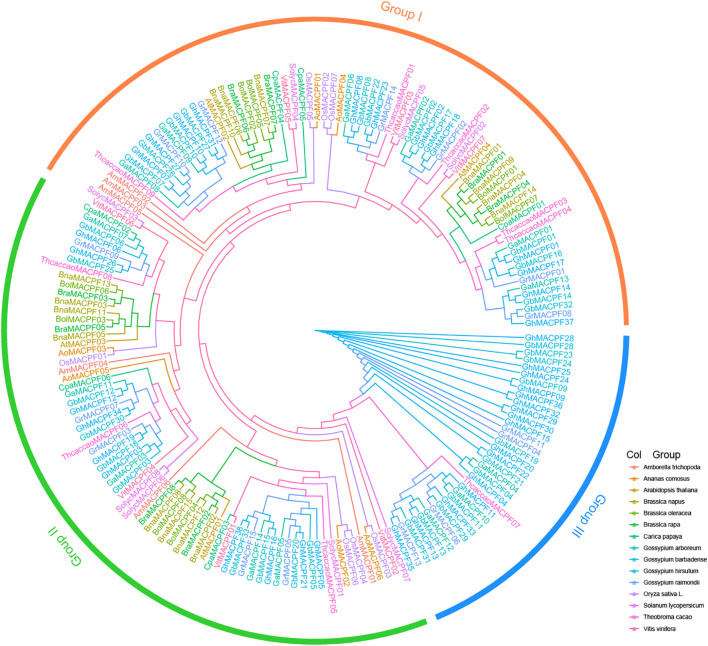
A phylogenetic tree of the membrane attack complex/perforin (*MACPF*) gene family. All the sequences of the *MACPF* domain gene in four *Gossypium* spp. and 11 other plants were carried out by using the maximum likelihood (ML) method in PhyML and members in the same species belong to the same color.

All the protein sequences annotated as the MACPF in four *Gossypium* and other 11 species were performed. A total of 184 MACPF domain-containing sequences were retained for this study (Supplementary Table 2). The number of the *MACPFs* is approximately similar to the genome size of the evolution biodiversity (Qiao et al., 2019). Similarly, the number of the *MACPF* genes in each allotetraploid is nearly equal to the sum of those in the two diploids. With the ML method and the conservative MACPF domain, the phylogenetic tree was constructed and classified into three branches (clades I–III) (Figure 1). However, the majority of the *MACPF* genes in group III (35 *MACPFs*) was specific and belonging to the Malvaceae, indicating that those genes likely experienced a sequence divergence event during polyploidization.

Basic information of the *MACPF* genes in *Gossypium* was listed in Supplementary Table 3 including genomic length, transcript length, GC count (%), exon number, molecular weight (MW), charge, and isoelectric point (PI). The genomic length of the *MACPF* genes was ranged from 790 (*GhMACPF22*) to 9,893 bp (*GrMACPF14*), the coding sequence (CDS) length of those range from 348 (*GhMACPF22* and *GbMACPF21*) to 1,872 bp (*GbMACPF19*), and the average GC content of the transcript was 43.13. Moreover, the exon numbers varied from 2 (*GhMACPF15*, *GbMACPF21*, and *GhMACPF22*) to 9 (*GbMACPF33*), and more than half of the *MACPFs* containing seven exons. The MW value ranged from 12.884 (*GbMACPF21*) to 69.677 kDa (*GbMACPF19*) and the PI values varied from 6.228 (*GhMACPF28*) to 9.889 (*GbMACPF15*) (Supplementary Table 3).

### Conserved Characteristics Analysis in the Diploid and Allotetraploid Cotton

The 2,000-bp upstream from the initiation codon in the diploid and allotetraploid cotton was used for the *cis*-element analysis of the *MACPF* genes. Through the PlantCARE web analysis, 15 *cis* elements were identified from the allotetraploid cotton, 14 *cis* elements in *G. arboreum*, and 13 *cis* elements in *G. raimondii*, respectively. Among those, 13 *cis* elements were common. Five kinds of hormone-response *cis*-elements, including ABA-responsive element (ABRE), auxin-responsive element (TGA-element), gibberellin (GARE-motif, P-box, and TATC-box), MeJA-responsive element (CGTCA-motif, and TGACG-motif), and SA-responsive element (SARE and TCA-element) were identified in each gene. Totally, four regulatory elements, which contained the low temperature responsive element (LTR), MYB-binding site (MBS), MYB-binding site I (MBSI), and TC-rich repeats, were found to have a certain function in the cold, drought, and defense response in plants (Supplementary Figure 1). Furthermore, the RY elements (seed-specific regulation) and circadian elements (*cis-*acting regulatory element involved in circadian control) were also found in the promoters, indicated that the *MACPF* gene might be played a role in plant development. The diversity analysis of elements determined the response of the *MACPFs* in *Gossypium* spp. to endogenous hormones, abiotic stresses, and development.

**FIGURE 2:**
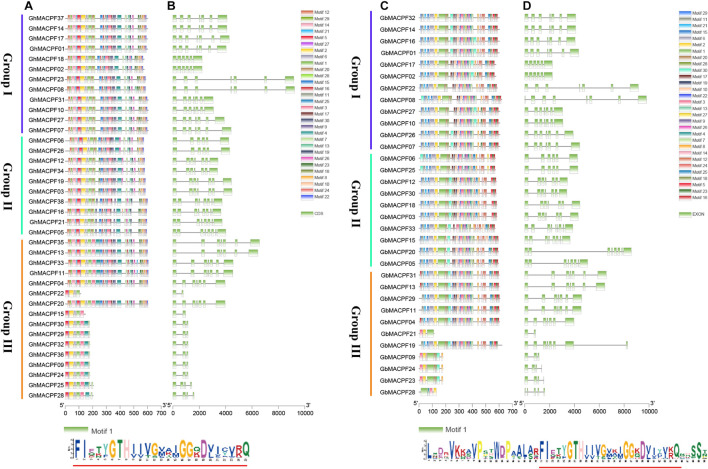
Motif and gene structure of the *MACPF* member in the allotetraploid cotton. **(A,C)** Are the converted motif and **(B,D)** are the gene structure of the MACPF member.

Combining the phylogenetic, motif composition, and gene structure analysis in Figure 2, the MACPF domain-contained proteins were divided into three groups. In this study, with the 30 distinct conserved motifs set through the MEME program, motif 1 was shared in *GhMACPFs* and *GbMACPFs* with the conserved “(F/Y)GTH(F/Y)-X6-GG” structure (Supplementary Figure 2). Moreover, motif 20 and motif 28 were specific in group I and group II and motif dispersion is variable in group III (Figure 2). Multiple motif distribution suggested that those genes were conserved and might have similar functions in the allotetraploid cotton. Moreover, the gene structure and intronic phase were exhibited similar to the MEME dispersion in the diploid, especially in group I and group II (Supplementary Figure 3). As described, the *MACPF* members were more likely to have gene selection and expansion between the diploid and allotetraploid cotton.

### Orthologous Genes Analyses Between the Diploid and Allotetraploid Cotton

**FIGURE 3:**
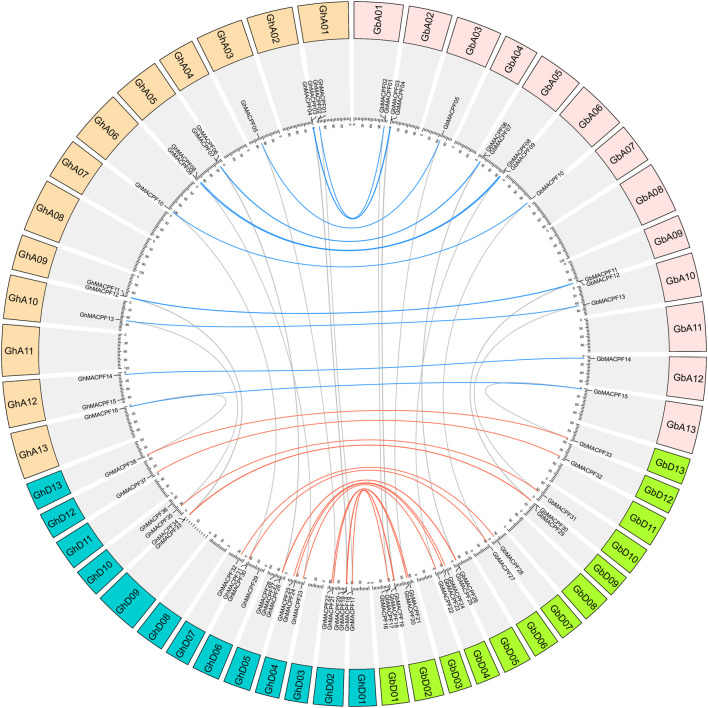
Duplication event among the *MACPF* members between *G*. *hirsutum* and *G*. *barbadense*. The Blue line indicates the homologous genes between the Gh_At and Gb_At subgenomes and the red line indicates the Gh_Dt and Gb_Dt subgenomes.

The chromosomal distribution of the *MACPF* genes in diploid cotton showed that 15 *GrMACPFs* were mapped to nine chromosomes of the *G*. *raimondii*. 14 *GaMACPFs* were also mapped to nine chromosomes of the *G*. *arboreum* (Supplementary Figure 4). A total of 38 *GhMACPFs* and 33 *GbMACPFs* were mapped to 19 chromosomes in the allotetraploid cotton, respectively. There was no *MACPF* gene located on chromosomes GhA04, GhA07, GhA08, GhA11, GhD03, GhD08, GhD11, GbA04, GbA07, GbA08, GbA11, GbD03, GbD08, and GbD11 (Figure 3). Numerous genes were varied from 1 to 4 on the mapped chromosomes (Figure 3, Supplementary Figure 4, and Supplementary Table 3). Duplication event analysis in the *MACPF* family indicated that there were 12 WGD/segmental events in Gh_At (75%), Gh_Dt (54.5%), Gb_At (80%), and Gb_Dt (66.7%); 9 WGD/segmental events (64.3%) in *G. arboreum*; and 11 events (73.3%) in *G. raimondii*, respectively. However, the singleton and proximal event were few in the *MACPF* family. It was suggested that the WGD/segmental was the main reason for the expansion of the *MACPF* gene family in *Gossypium* spp. (Supplementary Table 4).

Orthologous analysis showed 31 *MACPF* gene pairs in the two allotetraploid pieces of cotton including 14 gene pairs in Gh_At and Gb_At subgenomes and 17 pairs in Gh_Dt and Gb_Dt subgenomes. Meanwhile, 12 paralogous gene pairs were found in *G. hirsutum* and 14 paralogous gene pairs were found in *G. barbadense*, respectively (Figure 3 and Supplementary Table 5). Subsequently, orthologous genes were also identified between the two allotetraploid pieces of cotton and two diploids. A total of 26 *GhMACPFs* and 26 *GbMACPFs* were orthologous genes in the two diploid cottons of which 13 gene pairs showed in *G. arboreum*, while 13 gene pairs showed in *G. raimondii* (Supplementary Figure 4 and Supplementary Table 5). Furthermore, the Ka/Ks ratios for the multi-MACPF pairs in *Gossypium* spp. were determined in Gb_Dt-Gr, Gh_Dt-Gr, Gb_At-Ga, Gh_At-Ga, Ga-Gr, Gb_At-Gb-Dt, Gb_At-Gh-At, Gh_At-Gh-Dt, and Gb_Dt-Gh-Dt. Among the 121 gene pairs, the Ka/Ks ratio of *GbMACPF31-GhMACPF35*, *GbMACPF12-GaMACPF11*, and *GhMACPF12-GaMACPF11* were >1, the other 109 gene pairs were range from 0 to 0.94, indicating that these genes have undergone purifying selection (Supplementary Table 6).

### Expression Patterns and Co-expression of the Homologous *MACPF* Genes in the Allotetraploid Cotton

**FIGURE 4:**
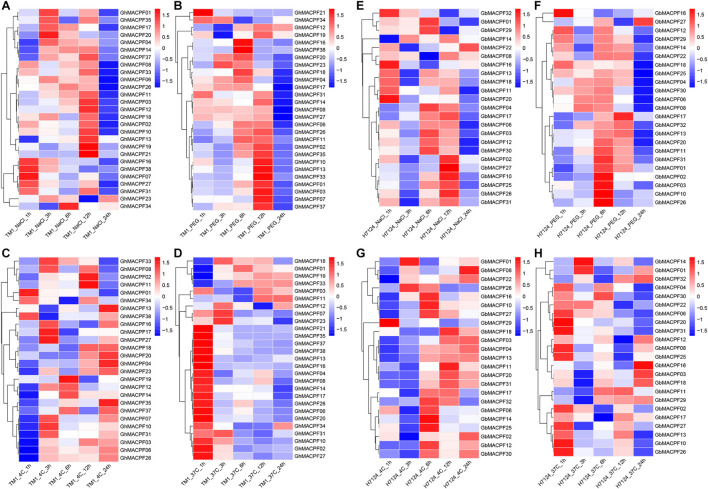
Expression profiles of the *MACPF* member in response to the different abiotic stresses. The expression levels of *GhMACPFs* and *GbMACPFs* under salt stresses **(A,E)**, polyethylene glycol (PEG) stresses **(B,F)**, cold stresses **(C,G)**, and hot stresses **(D,H)**, respectively.

**FIGURE 5:**
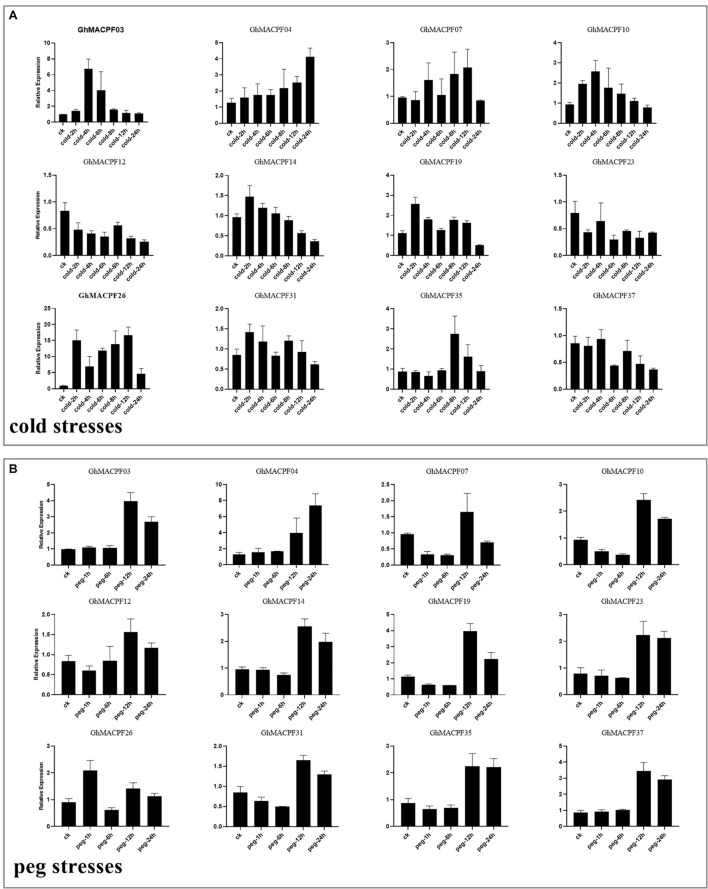
Expression levels of 12 *GhMACPF* genes in response to the **(A)** cold and **(B)** PEG treatment. Gene expression was analyzed by quantitative real-time-PCR (qRT-PCR). Error bars represent the SD of the three biological replicates.

To understand the potential function of *GhMACPFs* and *GbMACPFs*, we analyzed the expression patterns of the *MACPFs* in various tissues via the published RNA-seq data (Supplementary Figure 5 and Supplementary Table 7). Further, 27 *GhMACPFs* and 23 *GbMACPFs* were exhibited distinctive expression patterns in the different tissues. Most of the *MACPFs* were highly expressed in the anther, bract, filament, petal, pistil, and sepal (Supplementary Figures 5A,B). *GhMACPFs* and *GbMACPFs* were also highly expressed in the 5 DPA (day post anthesis), 10 DPA, and 15 DPA of the ovule, and low expression in fiber development stages (Supplementary Figures 5C,D). Different expression profiles of the *MACPFs* suggested that they widely participated in the various development stages of cotton. Furthermore, the expression pattern of the *MACPFs* in the allotetraploid cotton was regulated by cold, heat, salt, and drought stresses (Figure 4). Most of the selected *GhMACPFs* were predominantly expressed at 24 h under cold treatment, especially *GhMACPF04*, *GhMACPF13*, *GhMACPF26*, and *GhMACPF35*. We further carried out qRT-PCR to confirm the RNA-seq data after the PEG and cold treatments (Figure 5). The results suggested that *GhMACPFs* were involved in response to the cold and PEG stresses in cotton.

**FIGURE 6:**
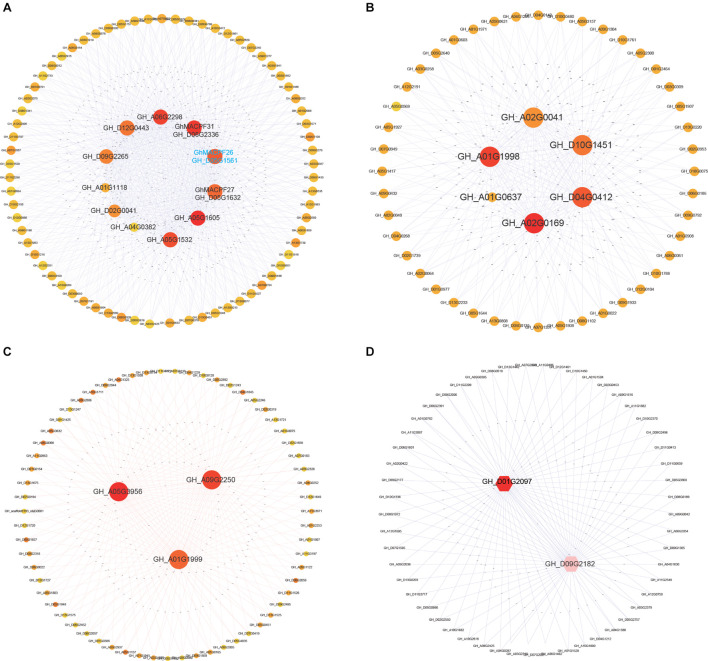
The correlation network of 19 *GhMACPF* members in cold stresses. All the gene networks are constructed by the weighted gene co-expression network analysis (WGCNA) in which each node represents a gene. **(A)** (nine *GhMACPFs*), **(B)** (five *GhMACPFs*), **(C)** (three *GhMACPFs*), and **(D)** (two *GhMACPFs*) indicate *GhMACPFs* in the different modules.

Co-expression analysis was constructed to uncover the potential interaction of functional genes with the *MACPFs* in cotton under cold treatment. Figure 6A displayed nine *GhMACPFs*, including *GhMACPF06*, *GhMACPF07*, *GhMACPF10*, *GhMACPF19*, *GhMACPF26*, *GhMACPF27*, *GhMACPF31*, *GhMACPF34*, and *GhMACPF39*, which might be co-expressed with the AP2, GRAS, VQ, WRKY, and C2H2 TFs, which are widely involved in cold stress response (Supplementary Table 8). These results were confirmed with qRT-PCR analysis (Supplementary Figure 6). Moreover, Figure 6B (*GhMACPF01*, *GhMACPF03, GhMACPF04*, *GhMACPF23*, and *GhMACPF35*), Figure 6C (*GhMACPF02*, *GhMACPF08*, and *GhMACPF11*), and Figure 6D (*GhMACPF18* and *GhMACPF33*) showed the other 10 *GhMACPFs* that were also participated in the cold responding interaction networks of TFs including AP2, p450, C2H2, basic helix-loop-helix protein (bHLH), and VQ domain-containing genes (Supplementary Table 8). *GhMACPFs* might interact with AP2, VQ, and WRKY TFs to enhance the cold resistance regulation of cotton.

### Silencing *GhMACPF26* Increased Tolerance to Cold Stress

**FIGURE 7:**
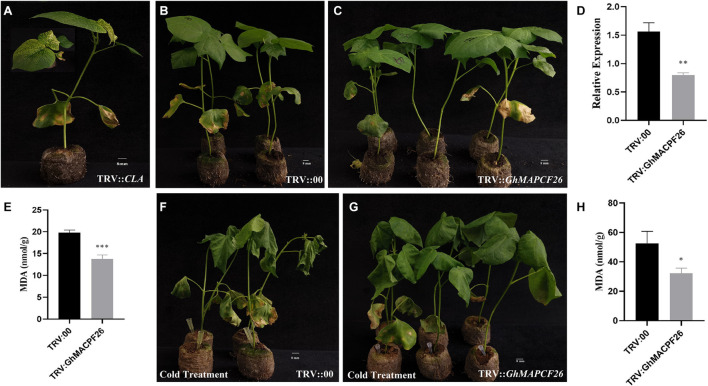
Silencing *GhMACPF26* via virus-induced gene silencing (VIGS) enhances the cold resistance regulation of cotton. **(A)** Plant albino phenotypes of TRV::*CLA*. **(B,C)** Phenotypes of TRV::00 and TRV::*GhMACPF26* before cold stress. **(D)** *GhMACPF26* expression levels in leaves of TRV::00 and TRV::*GhMACPF26* plants. **(E)** The malondialdehyde (MDA) content of TRV::00 and TRV::*GhMACPF26* before cold stress. **(F,G)** Phenotypes of TRV::00 and TRV::*GhMACPF26* after cold treatment for 24 h. **(H)** The MDA content of TRV::00 and TRV::*GhMACPF26* after cold stress. *p < 0.05, **p < 0.01, and ***p < 0.001 (Student’s one-tailed *t* test).

To investigate the role of *GhMACPF26* in response to cold stress, we performed a VIGS assay to decrease the *GhMACPF26* expression in TM-1 plants. The albino phenotype ensured the success of the tobacco rattle virus (TRV)::CLA1 in cotton (Figure 7A) and the comparison of the expression level of TRV::00 and TRV::*GhMACPF26* in cotton indicated that the gene expression had been successfully silenced (Figures 7B–D). TRV::*GhMACPF26* and TRV::00 plants were subjected to cold stress for 24 h. The empty control plants (Figure 7B) and TRV::*GhMACPF26* (Figure 7C) were similar before the cold treatment, but they contained MDA content that was significantly different based on the *t*-test analysis (Figure 7E). The phenotypic difference in the degree of leaf damage was clear between the TRV::00 (Figure 7F) and TRV::*GhMACPF26* (Figure 7G) after cold treatments for 24 h. The MDA content in TRV::00 plants was about 1.6 times more than in TRV::*GhMACPF26* content (Figure 7H). Additionally, the MDA content was examined to the degree of cell damage (Jouve et al., 1993). Our results preliminarily proved that silencing of the *GhMACPF26* gene improves cold tolerance in cotton.

## Discussion

Many MACPF domain-containing proteins were involved in the innate and adaptive immunity against pathogens through multiple pathways (Esser, 1994; McCormack et al., 2013; Spicer et al., 2017; Chen T. et al., 2020) and more and more pieces of evidence showed that the *MACPF* genes might participate in plant immune system, development, and abiotic stresses (Yu et al., 2020). In this study, 184 *MACPFs* were identified through a comprehensive analysis among the 15 genomes. Transcriptomic and co-expression analysis revealed that the *MACPF* genes were involved in the cold stresses, while silenced *GhMACPF26* enhanced cotton plant tolerance to cold stress. These results indicated that the *MACPF* genes might play an important role in cotton adaptation to abiotic stress.

### Phylogenetic, Duplication, and Structural Characteristics of the *MACPFs* in *Gossypium* spp.

The WGD or polyploidization is important for genome evolution due to the neofunctionalization and subfunctionalization of redundant genes (Blanc and Wolfe, 2004; Jiao et al., 2011; Qiao et al., 2019). Polyploidy on selection and domestication among the *Gossypium* spp. drives parallel gene expression in the development and abiotic stresses (Hu et al., 2019; Chen Z.J. et al., 2020). In this study, we found that the *MACPF* genes were shared with conserved gene numbers and similar duplication events by comparing the allotetraploid and diploid cotton. Totally, 38 *GhMACPFs*, 33 *GbMACPFs*, 14 *GaMACPFs*, and 15 *GrMACPFs* were identified from the four *Gossypium* spp. through a comprehensive gene family analysis, respectively. The number of the *MACPFs* in the allotetraploid cotton was almost two times the number in diploid cotton. The duplication event analysis showed that the WGD event was likely leading to the expansion of the *MACPF* genes in the allotetraploid cotton. Meanwhile, 184 *MACPF* genes were divided into three groups, of which 4 *AtMACPFs* and 7 *OsMACPFs* were evenly distributed in the Group I and Group II. The results showed that group I was conserved and belonged to the mallow (Figure 1). The conserved motif analysis in *Gossypium* spp. indicated that the “(F/Y)GTH(F/Y)-X6-GG” motif was widely distributed in *GhMACPFs*, *GbMACPFs*, *GrMACPFs*, and *GaMACPFs* (Figure 2 and Supplementary Figure 3). Orthologous analysis suggested that the evolution of the *MACPF* genes was not balanced between the allotetraploid and diploid genomes and there were more genes in the Dt subgenomes (Figure 3 and Supplementary Figure 4). The imbalance transcript indicated that the donors of the Dt subgenomes and *G. raimondii* were important to the abiotic stresses in the allotetraploid cotton and in the *Brassica napus* allotetraploids (Wang et al., 2019; Meng et al., 2020; Zhang et al., 2020). In addition, Ka/Ks values showed that most gene pairs have undergone purification selection during evolution (Supplementary Table 6) and the purification selection in WRKY and plant homeodomain (PHD) gene family (Hurst, 2002; Gu et al., 2018; Wu et al., 2021). These results suggested that the *MACPF* genes have been replicated in the ancient genome duplication events.

### Membrane Attack Complex/Perforins Play Important Roles in Abiotic Stress

*Cis*-regulatory elements within the promoter analysis are important to understand transcriptional regulation, which contained the development regulation and environmental responses (Chen et al., 2018; Yan et al., 2019). *Cis*-acting elements on the promoter were recruited and bound by TFs and the expression of the gene was regulated (Liu et al., 2013). As the previous results, the ABREs (ABA responsive elements) induced the expression of genes involved in the ABA signaling pathway (Hossain et al., 2010), while the GARE motif functions as a gibberellin-response element, TGA element acts as an auxin-response element, TGACG motif is the MeJA-response element (Chen and Qiu, 2020), and LTR is the low-temperature responsiveness element (Yamaguchi-Shinozaki and Shinozaki, 1994). ABRE, TGA, GARE- motif, CGTCA motif, TGACG motif, LTR, MBS, MBSI, and TC-rich repeats were also found in the *MACPF* promoters. These *cis* elements indicated that they participated in the different regulatory mechanisms of environmental adaptation including abiotic stress response (Supplementary Figure 1). Interestingly, the expression of the *GhMACPFs* and *GbMACPFs* widely responded to various abiotic stresses including the salt, PEG, cold, and heat treatments (Figure 4). In addition, most *GhMACPFs* and *GbMACPFs* were highly expressed in various tissues, and the ovule or fiber development, suggesting that the *MACPFs* also play an important role in the differentiation and fiber development of cotton tissue (Supplementary Figure 5). As reported in rice, maize, and *Arabidopsis* (Yu et al., 2020), *MACPF* genes were widely involved in abiotic stress response via the complex potential mechanism in *Gossypium* spp.

The Co-expression network was constructed to explore the potential connectivity of the genes involved in the plant development and abiotic stress response (Zhang and Horvath, 2005; van Dam et al., 2018; Chen P. et al., 2020; Cheng et al., 2020; Hu et al., 2020; Wang et al., 2020). TFs, including AP2/ERF, GRAS, VQ, and WRKY families, have recently been subjected to an intensive investigation because of increasing evidence of their response to abiotic stress (Mizoi et al., 2012; Grimplet et al., 2016; Gu et al., 2018; Chen P. et al., 2020). Constructing expression regulatory networks related to TFs is important for mining the candidate functional genes. Several *GhMACPFs*, including *GhMACPF6*, *GhMACPF7*, *GhMACPF10*, *GhMACPF19*, and *GhMACPF26*, were predicted to interact with AP2/ERF, WRKY, and VQ TFs (Figure 6 and Supplementary Table 8). The results indicated that the *MACPF* genes might act together with these TFs under cold stress in cotton.

Previous studies have shown that cold stress not only led to a decrease in growth and development but also affected plant metabolism and phytohormones (Thakur et al., 2010). Silencing *GhMACPF26* indicated that the VIGS plant enhances the cotton cold resistance via the measure of the MDA content (Figure 7). The MDA content in TRV::00 was significantly higher than the TRV::*GhMACPF26* after 24 h cold treatment, indicating that the plasma membrane damage is more serious in the TRV::00 plant (Figure 7). The MDA content is usually used to measure the degree of damage to plant cells and it also acts as the product of lipid oxidation (Leshem, 1987; Jouve et al., 1993). Transcriptomic results suggested that *GhMACPF* was also participating in the regulation of cotton tissue, ovule, fiber growth, and development. Moreover, we hypothesized that the *MACPF* members related to cotton resistance, especially resistance to cold stress. However, the underlying molecular mechanism requires further elucidation.

## Conclusion

In this study, a comprehensive analysis of the *MACPF* family was performed in the diploid and allotetraploid cotton via the phylogenetic, structural, orthologous, and transcriptomic analysis. Our data revealed that the WGD events might be the *MACPF* genes expansion in the allotetraploid cotton and genes on the Dt subgenome were related to stress resistance. *Cis* element, expression, and VIGS results indicated that the *MACPF* genes were activated in cotton response to cold stress. Co-expression analysis predicted that the *GhMACPF* genes might interact with AP2/ERF, WRKY, and VQ TFs to enhance the cold resistance of cotton. Silenced *GhMACPF26* increased the cotton tolerance to cold treatment. This study could provide new insights into the *MACPF* gene function in cotton and their potential interactions with other TFs.

## Data Availability Statement

The original contributions presented in the study are included in the article/Supplementary Material, further inquiries can be directed to the corresponding authors.

## Author Contributions

HW and SY designed the experiments. PC, HJ, and FW collected the sequences and analyzed the transcriptome analysis. AW, TH, XL, XG, JL, and LM participated in cotton culture processing and RNA extraction. LG, FW, and HW revised the language. PC performed the experiments and wrote the manuscript. All authors read and approved the final manuscript.

## Conflict of Interest

The authors declare that the research was conducted in the absence of any commercial or financial relationships that could be construed as a potential conflict of interest.

## Publisher’s Note

All claims expressed in this article are solely those of the authors and do not necessarily represent those of their affiliated organizations, or those of the publisher, the editors and the reviewers. Any product that may be evaluated in this article, or claim that may be made by its manufacturer, is not guaranteed or endorsed by the publisher.

## Footnotes

1
https://www.cottongen.org/

2
https://www.phytozome.net

3
https://www.ncbi.nlm.nih.gov/

4
https://pfam.xfam.org/

5
https://smart.embl-heidelberg.de/

6
https://www.ncbi.nlm.nih.gov/Structure/cdd/wrpsb.cgi

7
https://linux1.softberry.com/berry.phtml

8
https://web.expasy.org/translate/

9
http://phylogeny.lirmm.fr/phylo_cgi/index.cgi

10
http://meme-suite.org/

11
http://bioinformatics.psb.ugent.be/webtools/plantcare/html/
