# Supplementary figures and images for: Phylogenetic Analysis of the Membrane Attack Complex/Perforin Domain-Containing Proteins in Gossypium and the Role of GhMACPF26 in Cotton Under Cold Stress
Source: Front Plant Sci. 2021 Nov 19;12:684227. doi: 10.3389/fpls.2021.684227 (PMC8641546; doi:10.3389/fpls.2021.684227)

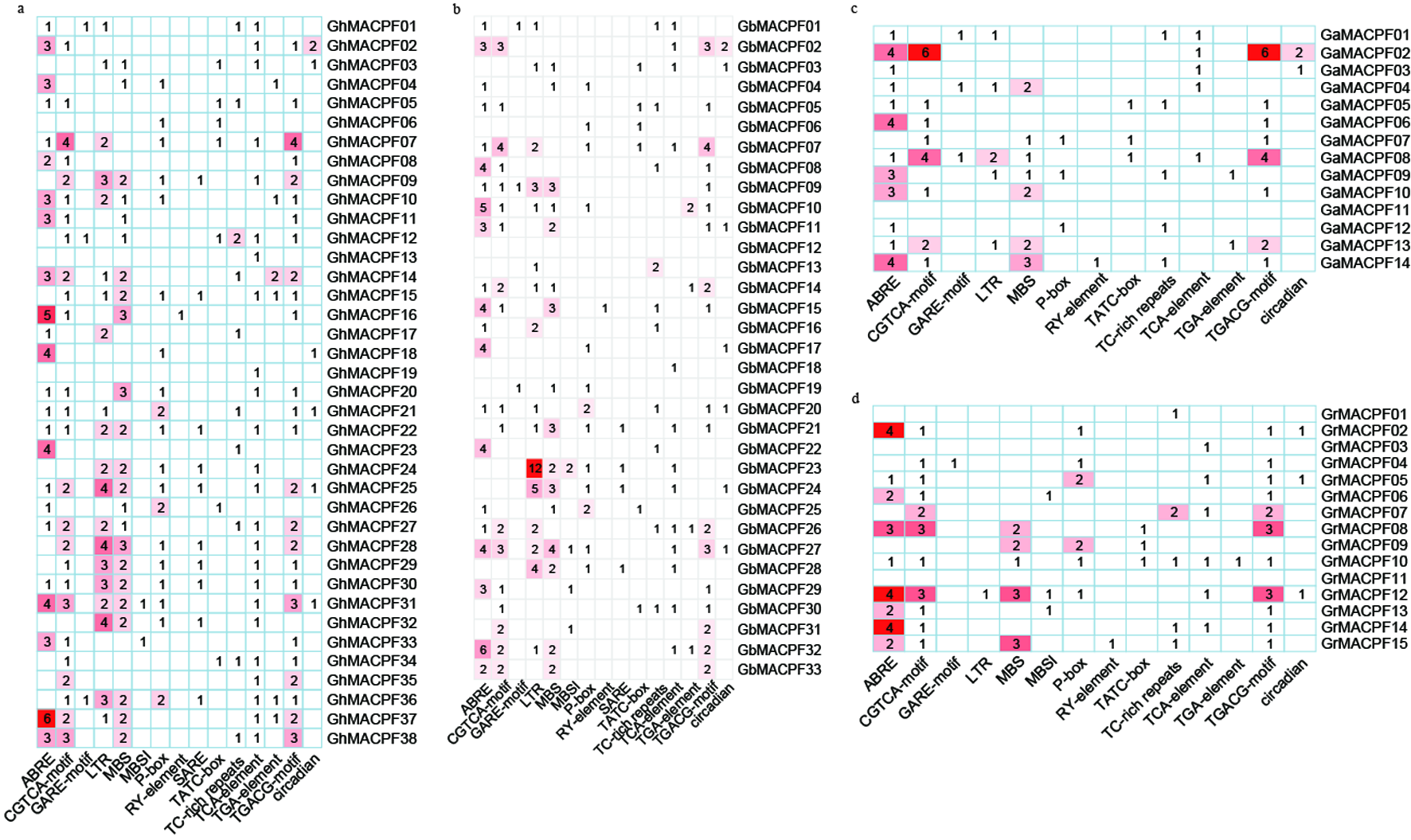

Supplement: Supplementary file 1 [file Image_1.TIF]

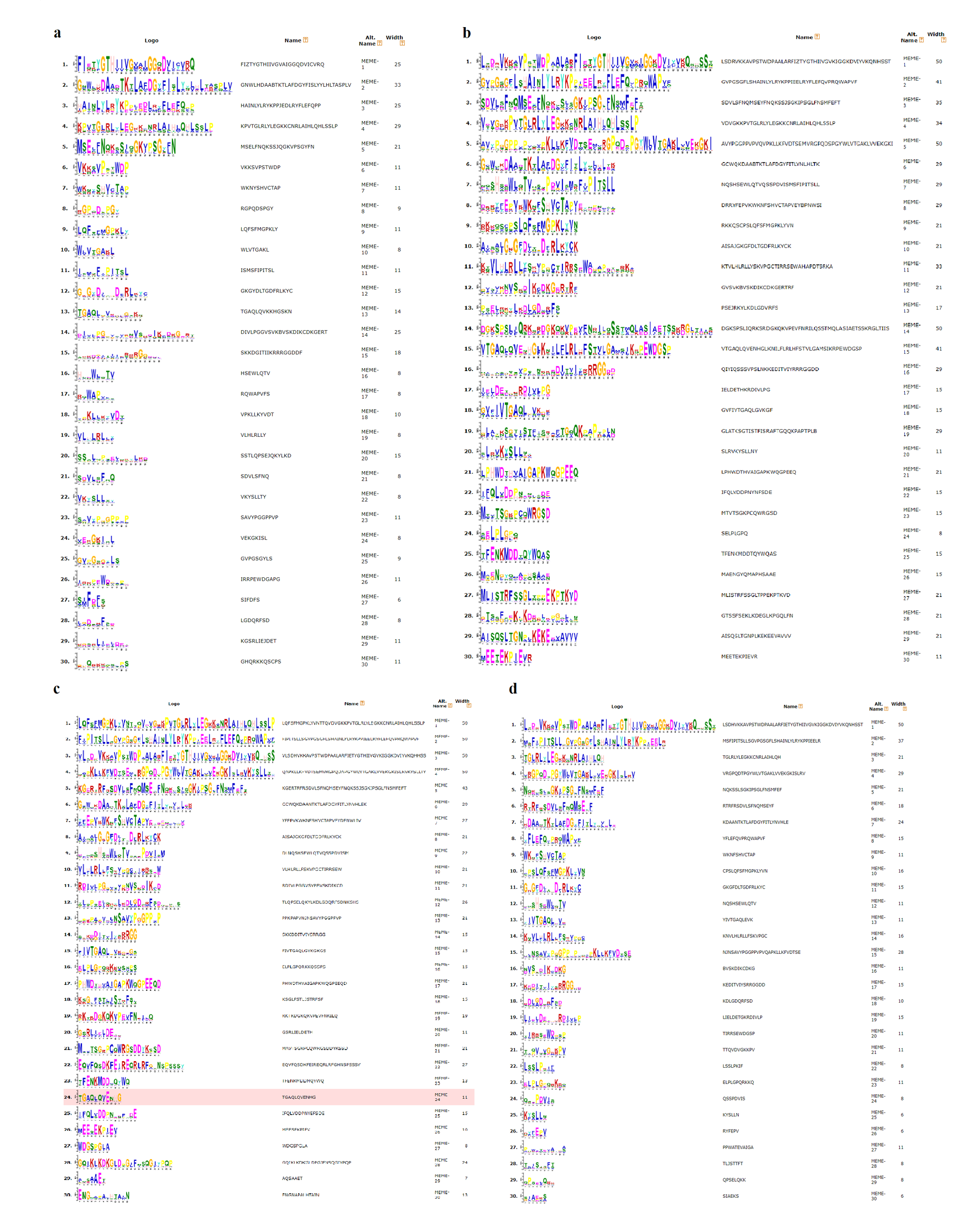

Supplement: Supplementary file 2 [file Image_2.TIF]

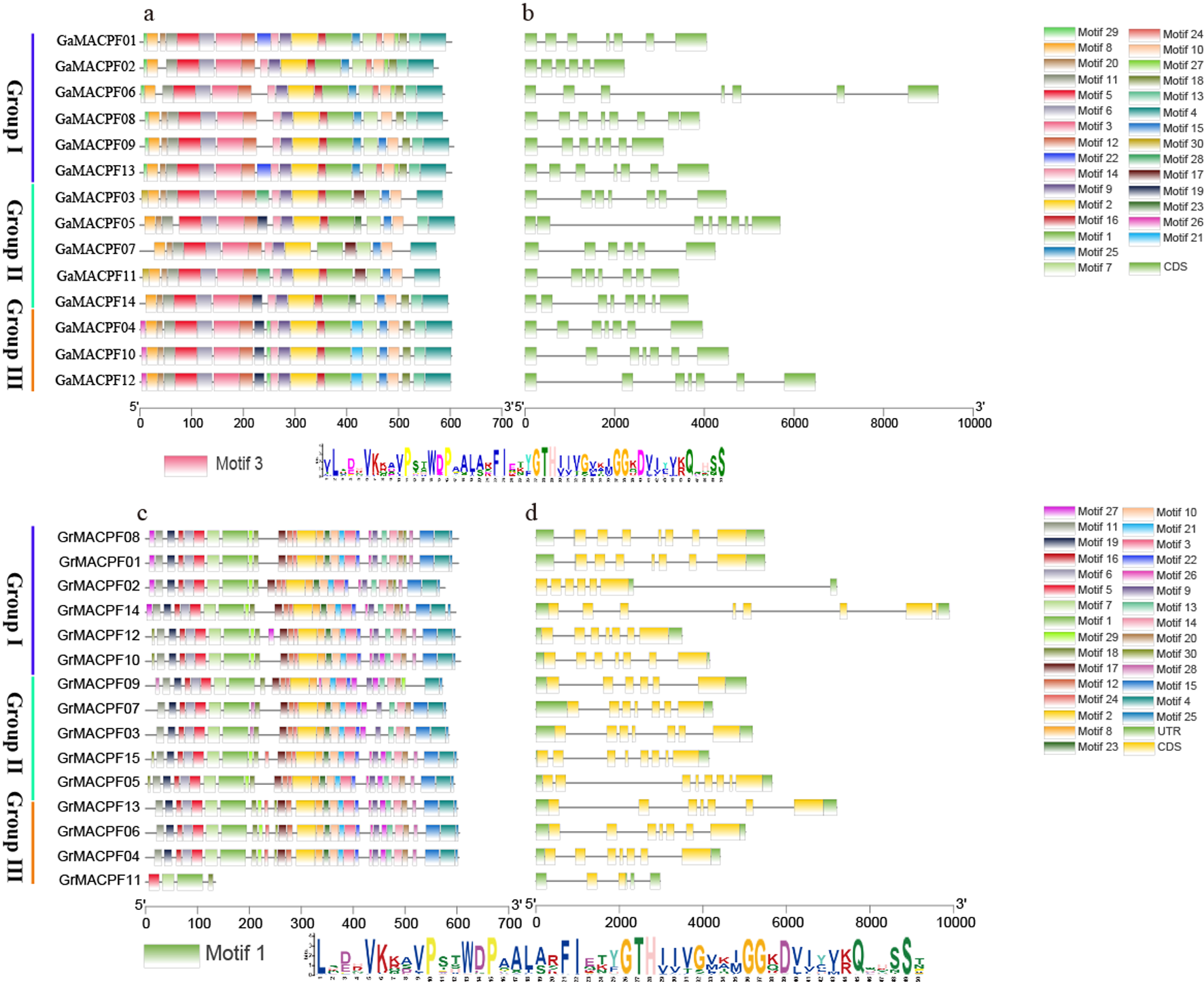

Supplement: Supplementary file 3 [file Image_3.TIF]

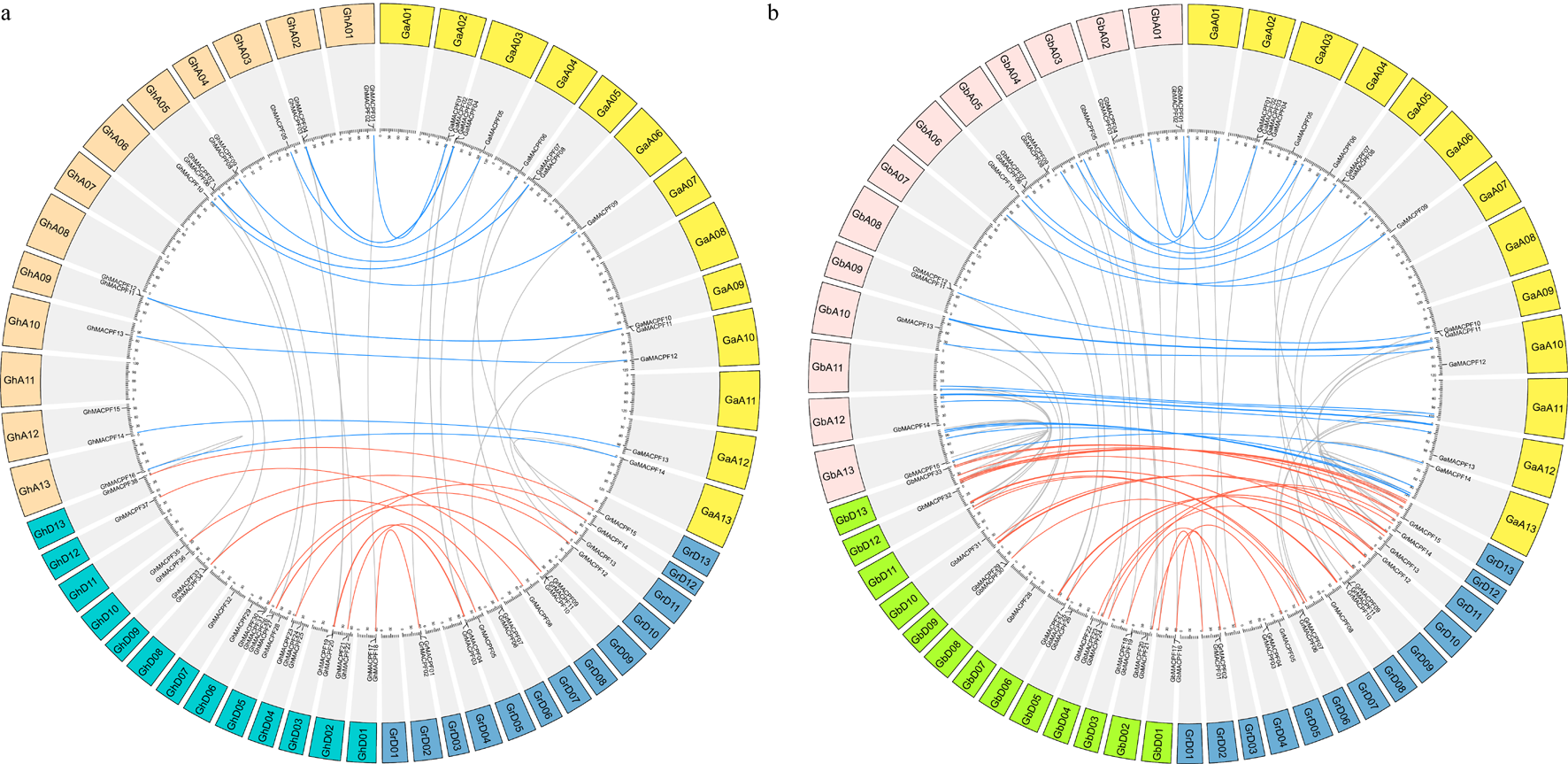

Supplement: Supplementary file 4 [file Image_4.TIF]

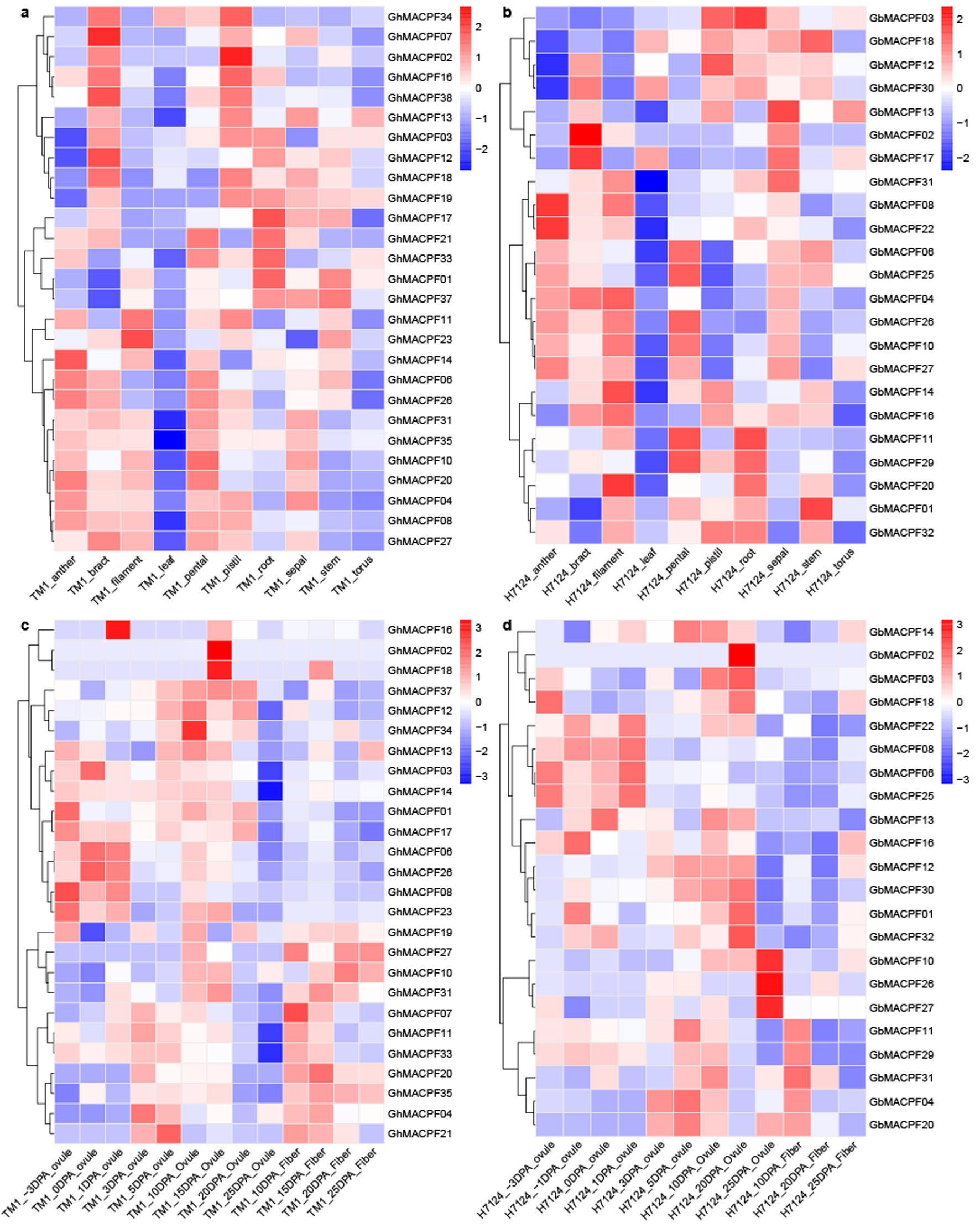

Supplement: Supplementary file 5 [file Image_5.TIF]

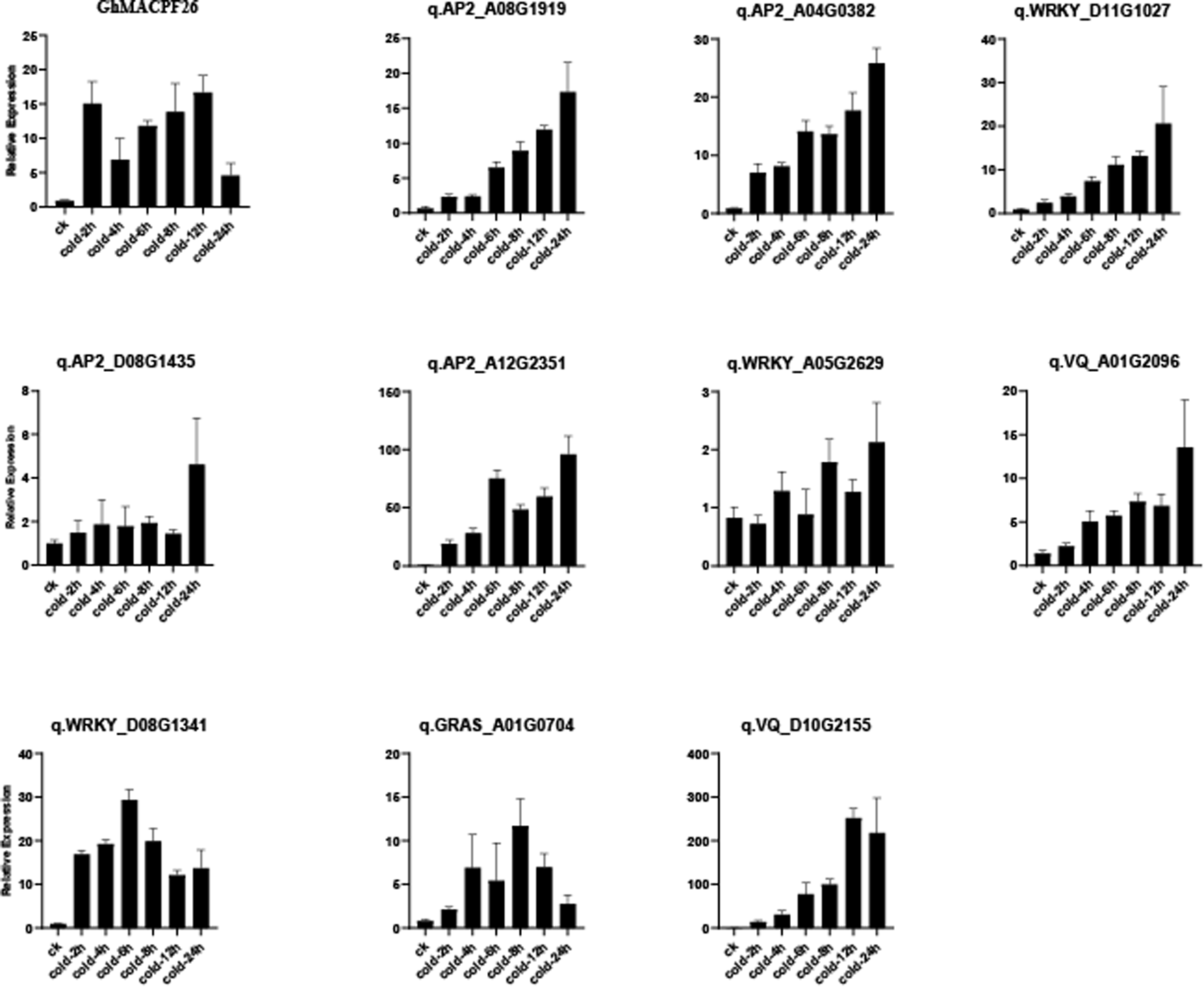

Supplement: Supplementary file 6 [file Image_6.TIF]
