# Supplementary material for: Phylogenetic Analysis of the Membrane Attack Complex/Perforin Domain-Containing Proteins in Gossypium and the Role of GhMACPF26 in Cotton Under Cold Stress
Source: Front Plant Sci. 2021 Nov 19;12:684227. doi: 10.3389/fpls.2021.684227 (PMC8641546; doi:10.3389/fpls.2021.684227)
Supplement: Supplementary file 8 [file Table_2.DOCX]

Supplementary Material

# Supplementary Data

Supplementary Material should be uploaded separately on submission. Please include any supplementary data, figures and/or tables. All supplementary files are deposited to FigShare for permanent storage and receive a DOI.

Supplementary material is not typeset so please ensure that all information is clearly presented, the appropriate caption is included in the file and not in the manuscript, and that the style conforms to the rest of the article. To avoid discrepancies between the published article and the supplementary material, please do not add the title, author list, affiliations or correspondence in the supplementary files.

# Supplementary Figures and Tables

For more information on Supplementary Material and for details on the different file types accepted, please see [here](http://home.frontiersin.org/about/author-guidelines#SupplementaryMaterial). Figures, tables, and images will be published under a Creative Commons CC-BY licence and permission must be obtained for use of copyrighted material from other sources (including re-published/adapted/modified/partial figures and images from the internet). It is the responsibility of the authors to acquire the licenses, to follow any citation instructions requested by third-party rights holders, and cover any supplementary charges.

## Supplementary Figures

**
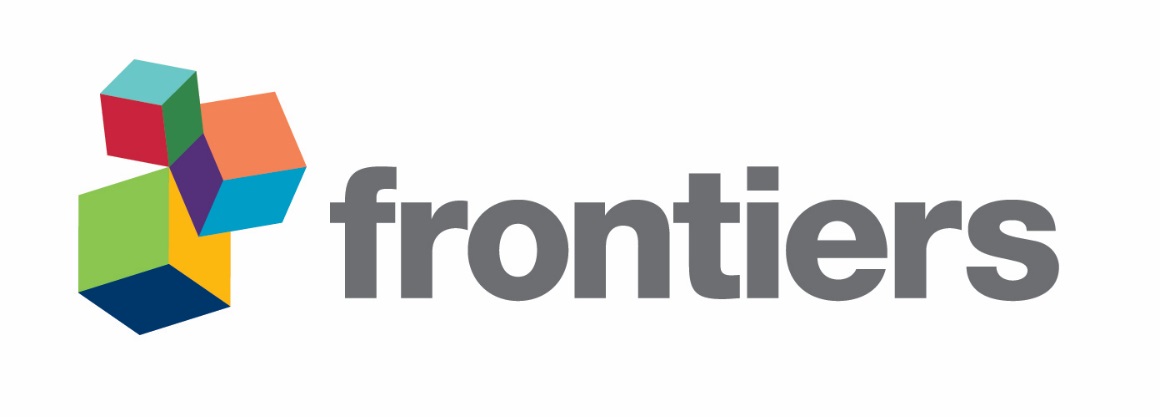
**

**Supplementary Figure S1.** *Cis*-elements analysis of the *GhMACPF* (a), *GbMACPF* (b), *GaMACPF* (c) and *GrMACPF* (d) promoters.

**Supplementary Figure S2.** Motif analysis of *MACPF* member in G. *hirsutum* (a), G. *barbadense* (b), G. *arboretum* (c), and G. *raimondii* (d).

**Supplementary Figure S3.** Motif, and gene structure of *MACPF* member in diploid cotton. a and c are the converted motif, and b and d are the gene structure of *MACPF* member.

**Supplementary Figure S4.** Duplication event among *MACPF* members between two diploid cotton and each allotetraploid cotton. Bule line indicate homologous genes between At and Ga subgenomes, and yellow line is the Dt and Gr subgenomes.

**Supplementary Figure S5.** Expression profile of *MACPF* member in various tissues, and fiber development. a and b The expression of *MACPF* genes in different tissues. c and d The expression levels of *MACPF* genes in ovule and fiber development.

**Supplementary Figure S6.** Expression levels of the AP2, WRKY, VQ and GRAS TFs in response to the cold stresses.

**Supplementary Table S1.** Primers used in this study.

**Supplementary Table S2.** Multi-fasta file the 184 MACPF protein sequences.

**Supplementary Table S3.** Summary of *MACPF* gene members in four Gossypium spp..

**Supplementary Table S4.** The duplicated type of *MACPF* genes in Gossypium spp.. The 0 to 4 indicate the singleton, dispersed, proximal, tandem, WGD duplication type, respectively.

**Supplementary Table S5.** The list of paralogous *MACPF* gene pairs in each of other examined species.

**Supplementary Table S6.** The Ka / Ks ratios for duplicated group *MACPF* genes in Gossypium spp..

**Supplementary Table S7.** The expression level of the *MACPF* genes in G. *hirsutum* and G. *barbadense*.

**Supplementary Table S8.** Co-expressed genes annotation by the NCBI / CDD database.
